# Coding-Complete Genome Sequences of Two Atypical Porcine Pestivirus Strains from Anhui Province, China

**DOI:** 10.1128/mra.00943-22

**Published:** 2023-01-04

**Authors:** Chunxiao Mou, Sihan Xie, Shuonan Pan, Zhenhai Chen

**Affiliations:** a College of Veterinary Medicine, Yangzhou University, Yangzhou, Jiangsu Province, People’s Republic of China; b Joint International Research Laboratory of Agriculture and Agri-Product Safety, Ministry of Education of China, Yangzhou University, Yangzhou, Jiangsu Province, People’s Republic of China; c Jiangsu Co-Innovation Center for Prevention and Control of Important Animal Infectious Diseases and Zoonoses, Yangzhou University, Yangzhou, Jiangsu Province, People’s Republic of China; Katholieke Universiteit Leuven

## Abstract

In 2021, two atypical porcine pestivirus (APPV) strains, AH06/2021 and AH22/2021, were identified from suckling piglets showing congenital tremor in Anhui Province, China. Genome sequence analysis indicated that the two strains shared 81.19% to 93.98% nucleotide identities with other APPV strains.

## ANNOUNCEMENT

Atypical porcine pestivirus (APPV) is a member of the genus *Pestivirus* of the family *Flaviviridae*, with a positive-sense and single-stranded RNA genome (1). APPV was first discovered in North America in 2015 using metagenomics (2). Since then, APPV has been detected in domestic pig populations all over the world, including many countries in Europe (3, 4) and Asia (5, 6). The presence of APPV in piglet-producing farms is associated with congenital tremor (CT) type A-II in newborn piglets and leads to so-called “shaking piglets” (7, 8). APPV can induce neurological disorders, reduce reproductive performance, and increase preweaning mortality rates, resulting in economic losses in piglet production (3, 9).

Piglet-producing farms located in the Anhui Province, China, have sporadically displayed cases of CT in suckling piglets. Serum, brain, liver, heart, spleen, lung, kidney, lymph node, and tonsil samples from four 10-day-old piglets with CT symptoms were collected for pestivirus examination in April 2021. These tissues were transported at 4°C and then homogenized with an electric homogenizer after the addition of ice-cold Dulbecco’s modified Eagle’s medium (DMEM). The homogenized mixture was centrifuged at 12,000 × *g* for 10 min at 4°C, and the supernatant was then collected. RNAs from these tissue samples were prepared using the High Pure viral RNA kit (Takara, Beijing, China), following the manufacturer’s protocol. The cDNA synthesis was performed using the PrimeScript II first-strand cDNA synthesis kit (Takara). Subsequently, 7 pairs of primers based on the CH-GX 2016 strain (GenBank accession number KY652092) were designed to amplify the coding-complete genome of APPV (Table 1). A total of 7 overlapping fragments were amplified by reverse transcription (RT)-PCR using PrimeSTAR DNA polymerase (Takara). The PCR amplicons were purified, cloned into a pMD19-T vector, and sequenced in both directions with an ABI 3730XL sequencer (Takara). The sequences of the 7 fragments were assembled into the coding-complete genomes for these APPV strains using Lasergene version 7.10 (DNASTAR, USA), and open reading frames (ORFs) were determined by comparison with the APPV GX-GL1 strain (GenBank accession number MN584738.1).

**TABLE 1:** Primers used in the RT-PCR for detection of APPV and amplification of the coding-complete virus genomes^*a*^

Primer	Primer sequence (5′ to 3′)	Target nucleotide positions
A1-F	GCATTATGTGTGGGACATCCTAA	21–1635
A1-R	CTTACACCCTGTCAGTGGACTCACC	
B-F	AGTCTGGGAATGATAGATACAAGC	1408–2725
B-R	CATTGTCACCGGGCCAATAGGG	
K-F	TAGTCAAAATATCAAAGGGGAACCT	2409–3811
K-R	TTCAAGTTTACATGGGGTGAAATC	
M-F	CGCCGATTTCACCCCATGTAAACTTG	3807–5343
M-R	CTGGTCTTGACTTTCAGTGCATGGATC	
G-F	GTCACAATAAAGGAGGGCAAAGTG	9100–10974
G-R	CTTCCAGATCCCTAAGTAAGTC	
N-F	GATGAACTATAGCATGACTTACTTAGG	10959–11142
N-R	CGTATTACTCCATTCATTCAAGTATTTACAAC	
I-F	CTCAGGTCATTCGTAGGTGGGAAG	11123–11511
I-R	TGCTTCATCTAGATCGGACTTATAC	

a Strain GX-CH 2016 (GenBank accession number KY652092) was used as the template for primer design.

The genome sequences of APPV strains AH06 and AH22, which were from the same farm but different piglets, were determined to be 11,194 nucleotides in length and contain a single ORF encoding a polyprotein comprising 3,635 amino acids, a 5′ untranslated region (UTR), and a 3′ UTR. The genomic G+C contents of the sequenced APPV strains AH06 and AH22 were 46.49% and 46.56%, respectively. The polyprotein was composed of 12 proteins, including four structural proteins (C, E^rns^, E1, and E2) and eight nonstructural proteins (N^pro^, P7, NS2, NS3, NS4A, NS4B, NS5A, and NS5B). The ORF sequence alignments revealed that strain AH06/2021 shared 82.07% to 93.93% nucleotide identities with the APPV strains that were recently isolated in China, the United States, and Austria, while it displayed the greatest similarity (93.93%) to the APPV GX-GL1 strain (GenBank accession number MN584738.1). Furthermore, sequence analysis demonstrated that the N^pro^, E2, and NS3 genes of APPV AH06/2021 shared 86.59% to 95%, 95.02% to 99.0%, and 73.94% to 98.98% nucleotide identities, respectively, with those of other APPV strains, while the N^pro^, E2, and NS3 genes of APPV AH22/2021 showed 86.59% to 95%, 95.02% to 98.64%, and 73.8% to 99.27% nucleotide similarities, respectively, to those of other APPV strains. In comparison, the APPV AH06/2021 and AH22/2021 strains displayed the greatest similarity to each other, with 99.7% to 99.8% nucleotide identities in the N^pro^, E2, and NS3 genes. The sequences of the two APPV strains will facilitate future research on the epidemiology and evolutionary biology of APPV in China.

### Data availability.

The complete genome sequences of the two APPV strains (AH06/2021 and AH22/2021) have been deposited in GenBank under the accession numbers OL625611.1 and OL625612.1. The BioProject accession number is PRJNA892274.
